# Establishment of an Efficient *In Vitro* Regeneration Protocol for Rapid and Mass Propagation of *Dendrobium chrysotoxum* Lindl. Using Seed Culture

**DOI:** 10.1155/2014/740150

**Published:** 2014-10-20

**Authors:** Potshangbam Nongdam, Leimapokpam Tikendra

**Affiliations:** Department of Biotechnology, Manipur University, Canchipur, Imphal, Manipur 795003, India

## Abstract

An efficient *in vitro* regeneration protocol from seed culture has been established successfully for *Dendrobium chrysotoxum*, an epiphytic orchid having tremendous ornamental and medicinal values. Seed germination response was encouraging in Mitra (M) medium enriched with different combinations of auxins and cytokinins. Medium supplemented with 0.4% activated charcoal (AC), 2 mg/L 6-benzyl amino purine (BAP), and 2 mg/L indole-3-acetic acid (IAA) produced best seed germination percentage in 2 weeks of culture. Incorporation of higher concentration of kinetin (KN) or BAP in combination with low auxin in medium induced pronounced shooting and leaf formation. Reduction in leaf development was evident when cytokinins exist singly in medium indicating synergistic effect of auxin and cytokinin in leaf induction. Presence of elevated level of indole-3-butyric acid (IBA) or 1-naphthalene acetic acid (NAA) with low cytokinin content in medium generated more *in vitro* rooting, though IBA was found to be more effective in rooting induction as compared to NAA. The *in vitro* protocol for asymbiotic seed germination developed from the present investigation can be used for rapid mass propagation of this highly important *Dendrobium* orchid species.

## 1. Introduction

*Dendrobium chrysotoxum* belongs to the family Orchidaceae which is considered as one of the largest and most advanced angiosperms consisting of more than 25000 species. The species is sympodial in growth habit and is mostly found at an elevation of 700 to 1200 m in deciduous forests of Northeast India, Bhutan, China, Thailand, and Laos.* D. chrysotoxum* known commonly as “Golden orchid” is extremely popular in local floriculture market due to its arching inflorescence besotted with 15–20 highly attractive honey fragrant yellowish bright coloured flowers. Apart from its high floricultural appeal, the species is widely known for medicinal values as it possesses antioxidant and antitumor properties. The polysaccharides obtained from the stem of* D. chrysotoxum* induced antioxidative, hypoglycemic, and immune stimulant effects in mouse systems [1]. Erianthridin extracted from the plant also produced anti-inflammatory activities [2]. They are employed as herbal drug in traditional system of medicine by indigenous people of Northeast India for treatment of various local ailments. The liquid extract obtained from the boiled leaves of* D. chrysotoxum* is used as tonic and antipyretic [3]. The thriving population of this multiutility orchid has witnessed a sharp decline in recent years due to various unwanted human activities. Excessive collection of orchid for illegal trade and rampant destruction of natural habitat for traditional agricultural practices and industrial expansion are the main reasons for the rapid reduction of orchid population. Plants belonging to Orchidaceae are all listed in International Union of Conservation of Nature (IUCN) Red Data Book. In fact* D. chrysotoxum* has been recently included in Appendix II of nearly threatened species of plants and animals under CITES (Convention of International Trade in Endangered Species of Wild Fauna and Flora) [4]. Effective conservation strategies should be devised to prevent further loss of the already depleted orchid population. The* in vitro* approach through the application of plant tissue culture technology provides an excellent opportunity for effective conservation by mass propagating orchids in short time span. However, the use of micropropagation techniques for propagation of* D. chrysotoxum* at commercial scale has not been fully realised due to lack of efficient and reliable protocols for seed germination, very limited understanding of culture growth and development* in vitro* and high mortality rate during hardening process, and final transplantation to field conditions. Ever since the first reports of* in vitro* asymbiotic seed germination in* Laelia-Cattleya* by Knudson [5] and shoot-tip culture in* Cymbidium* by Morel [6], several orchid species had been successfully propagated* in vitro* using different explants such as leaves, flower inflorescence, roots, and pseudobulb [7–10]. Though the available literature reveals the accomplishment of many investigators to propagate orchids* in vitro*, there are fewer reports on* in vitro* culture of* D. chrysotoxum* using asymbiotic seed culture. Orchid seeds which are extremely light, small, and nonendospermic cannot be used directly for mass propagation as very few seeds germinate independently in nature [11]. The germinated seeds will not grow further unless infected by a suitable mycorrhizal fungus [12]. The association of orchid seeds with appropriate fungus is essential to augment the physiochemical stimulus essential for seed germination and further development [13].* In vitro* seed germination using plant tissue culture techniques will circumvent this limitation by providing necessary inorganic and organic nutrients for seed germination. However, the* in vitro* orchid seed germination under asymbiotic condition is not always successful due to presence of thick cuticle which lowers the seed germination potential but protects the thin walled miniature seeds physically [14]. Concerted efforts and focussed investigation in this line will help in devising highly efficient and commercially viable micropropagation techniques of orchid seed culture for rapid and mass propagation of quality planting materials. The present study was carried out with an objective of establishing an efficient* in vitro* regeneration protocol for large scale propagation and effective conservation of this highly prized orchid species through seed culture.

## 2. Materials and Methods

### 2.1. Plant Source and Surface Sterilization

The 4-month old unripe green capsules of* Dendrobium chrysotoxum* Lindl. were used as explants to initiate culture. The collection of capsules was done from Khonghampat Orchid Centre and forested areas of Senapati District, Manipur, in Northeast India during November-December 2012. The capsules were first washed properly in running tap water with 20% teepol for 10–15 minutes which was followed by surface sterilization of capsules by treating them in 0.4% HgCl_2_ solution for 7-8 minutes. The capsules were washed 4-5 times in sterile double distilled water to remove HgCl_2_ completely from its surface. The capsules were finally flamed for 2-3 seconds after dipping in 70% ethyl alcohol for 8–10 minutes. The capsules were split open longitudinally by using sterile scalpel to scoop out numerous minute exalbuminous seeds and spread out on the culture media in sterile condition under laminar hood.

### 2.2. Media Preparation, Hormonal Combinations, and Incubation

Mitra et al. [15] medium was prepared to grow the capsule derived seeds of* D. chrysotoxum* and study the* in vitro* germination and growth response. The Mitra medium contained potassium and calcium nitrate as chief sources of nitrate while sulphate and phosphate were provided by magnesium sulphate and sodium phosphate, respectively. Separate stock solutions of macro- and micronutrient components of Mitra medium, vitamins, hormones, and iron salts were prepared in a concentrated solution and stored at 25–30°C until use. The macro- and micronutrients were added one by one as required with thorough mixing from stock solutions to a conical flask of desired size and volume makeup was done with double distilled water as per requirement before adjusting the pH at 5.8. Sucrose (2%) was added as carbohydrate source and 0.9% agar was used as a gelling agent. Growth medium was incorporated with 0.4% of AC. The AC was completely dissolved by swirling the vessels containing the boiled medium before the medium gets solidified. The plant growth regulators were added into the medium and the culture vessels were made air tight by closing with appropriately sized cotton plugs after definite quantities of medium were dispensed to culture vessels. The medium was autoclaved finally at 121°C for 15 minutes and placed in an appropriate position either vertical or slanting to allow the medium to gel. The culture medium was supplemented with indole-3-butyric acid (IBA), indole-3-acetic acid (IAA), 1-nathyl acetic acid (NAA), 6-benzyl amino purine (BAP), and kinetin (KN) in varied combinations and concentrations to study their influence on growth and development of culture* in vitro*. The range of plant growth hormones concentration for BAP (0.5 mg/L, 1.8 mg/L, 2.0 mg/L, 2.5 mg/L, and 3.5 mg/L), KN (1 mg/L, 1.5 mg/L, 2.0 mg/L, 2.5 mg/L, 3.0 mg/L, and 3.5 mg/L), IBA (0.5 mg/L, 0.8 mg/L, 1.0 mg/L, 1.5 mg/L, and 3.0 mg/L), IAA (0.5 mg/L, 1.0 mg/L, and 2.0 mg/L), and NAA (0.5 mg/L, 1.5 mg/L, 2.5 mg/L, and 4.5 mg/L) had been used for the present study. The cultures after inoculation onto the medium having different hormonal combinations were maintained at 25 ± 2°C with proper light illumination at 60 *μ*molm^−2^ s^−1^ for 12 hours a day using white fluorescent tubes.

### 2.3. Hardening of* In Vitro* Raised Plantlets

The process involved the transfer of well grown seedlings with fully developed leaves and roots to full strength culture medium devoid of any plant growth regulators and cultured them for 2-3 weeks. The sucrose and vitamins were removed from culture medium and the plants were grown in the same condition for another 2-3 weeks. The well rooted seedlings with healthy leaves were taken out from culture vessels using sterile forceps and transferred to culture flasks containing sterile bricks and charcoal pieces along with thinly spread coconut husks. The plantlets were grown for 3-4 weeks after which they were removed and treated with 0.01% fungicide solution for 15–20 minutes to remove fungal contaminants if any in seedlings. The plants were then transplanted to small plastic/clay pots containing brick pieces, pine bark, charcoal pieces, and moss (1 : 1 : 1) as potting mixture. The transplanted plants were finally kept in glass house for further acclimatization to nursery condition.

### 2.4. Data Recording and Statistical Analysis

The experiments were conducted thrice using eight replicates per treatment. The first subculture was performed in 10 weeks after which subculturings to freshly prepared medium were done after every 3 weeks. The culture responses with regard to callus formation, shooting, and rooting development were recorded at regular interval. Seed germination response was also examined by recording the germination percentage on different growth hormone combinations. The seed germination percentage was derived by using the following formula:

(1) Seed  germination  percentage=Number  of  seeds  successfully  germinated  by  swellingTotal  number  of  seeds  inoculated×100.

The data obtained from the present investigation were subjected to analysis of variance (ANOVA) and significant differences were determined by employing Duncan's multiple range test at *P* = 0.05 [16]. The statistical data analysis was performed using SPSS software program (SPSS Inc., Chicago, USA).

## 3. Results and Discussion

**Figure 1:**
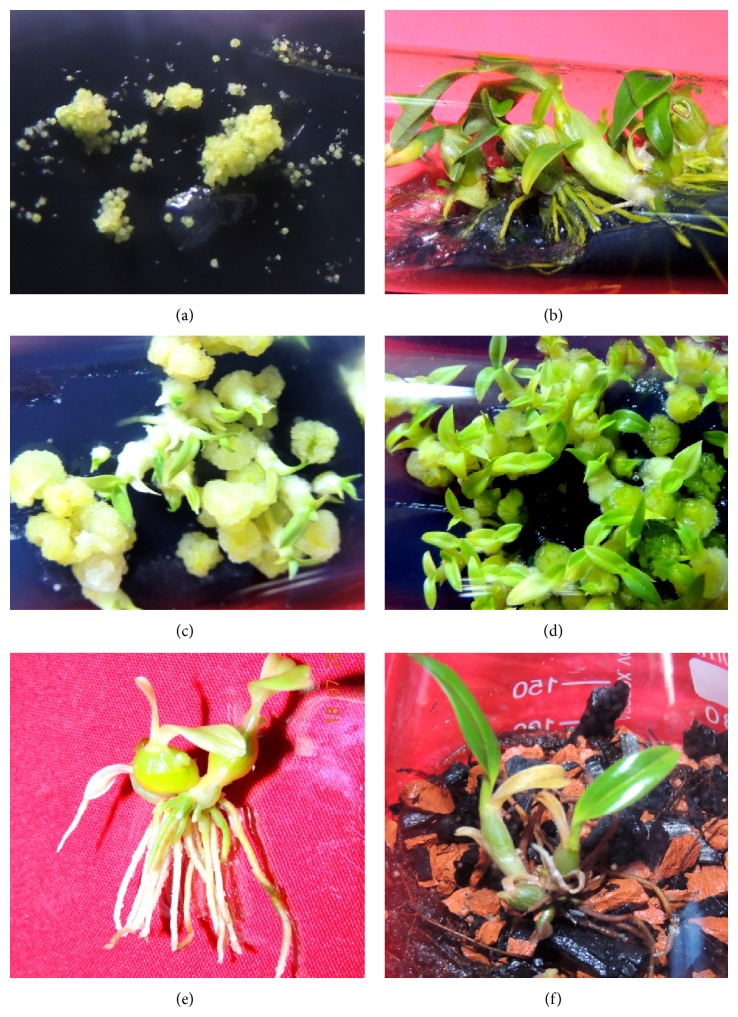
*In vitro* regeneration of* Dendrobium chrysotoxum* through seed culture. (a) Seed swelling indicating successful* in vitro* germination on medium enriched with 2.5 mg/L BAP and 0.8 mg/L IAA. (b)* In vitro* development of seedlings complete with healthy leaves and roots. (c) Intense callus formation in medium augmented with 2.5 mg/L BAP and 0.8 mg/L IBA. (d) Rapid leaf multiplication in medium supplemented with 2.0 mg/L KN and 0.5 mg/L NAA. (e) High* in vitro* root proliferation after seedlings transferred to new medium with 0.5 mg/L BAP and 3.0 mg/L IBA. (f) Hardening of* in vitro* raised healthy plantlets in basal liquid medium containing charcoal and brick pieces along with fine threads of coconut husks.

Seed germination was tested on AC (0.4%) incorporated basal medium as well as different plant growth regulators supplemented medium at varied concentrations. There was variation in seed germination response in regard to plant growth regulator combinations and concentrations. The exalbuminous orchid seeds because of limited food reserves need specific nutritional requirement under certain environmental conditions [17]. The nutritional requirement and seed germination stimulus are provided by inorganic and organic constituents of culture medium along with different additives and exogenous plant growth regulators. The same was true for* D. chrysotoxum* seeds in the present study when immature seeds were inoculated on medium augmented with different growth regulators at varied concentrations. Xu et al. [18] also described the importance of plant growth regulators, nitrogen source, and organized elements for seed germination and culture development in* D. chrysotoxum*. The seeds started swelling 2 weeks after inoculation in every hormonal combination tested indicating successful germination (Figure 1(a)). The enlargement of nonendospermous seeds was due to absorption of water and nutrients from culture medium and this phenomenon was observed in all orchid seeds which underwent successful* in vitro* germination [19]. Medium supplemented with AC, 2.0 mg/L BAP, and 2.0 mg/L IAA produced highest seed germination percentage (98.1 ± 3.9) while the least was observed in basal medium without any plant growth regulators (Table 1). The increase in seed germination with BAP in medium was earlier reported in* Cypripedium* [20]. Nongdam and Chongtham [21] also observed significantly high seed germination rate in* C. aloifolium* when MS medium was enriched with BAP and 0.2% AC. The lower germination response in basal medium in the present study might probably be due to absence of plant growth regulators which can exert positive influence on seed germination. The seed germination promoting nature of plant growth regulators has been previously described in several other orchid species [22–24]. The germinated seeds differentiated into spherical shaped protocorms via spherules formation. The protocorms turned green in coloration as chlorophyll pigment emerged in course of development from initial irregularly shaped spherules to protocorm bodies. The chlorophyllous protocorms underwent morphogenetic changes leading to pseudobulb and leaf primordial formation which subsequently developed into complete seedlings with leaves and roots in 10 weeks (Figure 1(b)). However, all the germinated seeds did not give rise to seedlings due to intervening callus formation in all the combinations tested. Though MS + 2 mg/L BAP + 2 mg/L IAA produced highest seed germination percentage (98.1 ± 3.9), only 91.63 ± 3.7% of the germinated seeds developed into complete plantlets successfully. The inability to generate seedlings from every germinated seed might be due to morphogenetic differentiation of some of the protocorms into callus tissues. Maximum seedling conversion (92.66 ± 3.2) from germinated seeds was recorded in medium incorporated with 1.5 mg/L KN and 1.5 mg/L IBA. Incidence of intense callus formation was observed in M + 2.5 mg/L BAP + 0.8 mg/L IBA which was the reason for the marginal success in seedling conversion (82.22 ± 4.3) of germinated seeds even though high seed germination percentage (96.1 ± 4.3) was recorded (Figure 1(c)). Similar observation of high callus induction in presence of BAP was previously reported in* Dendrobium chrysotoxum* by Roy et al. [25].

**Figure 2:**
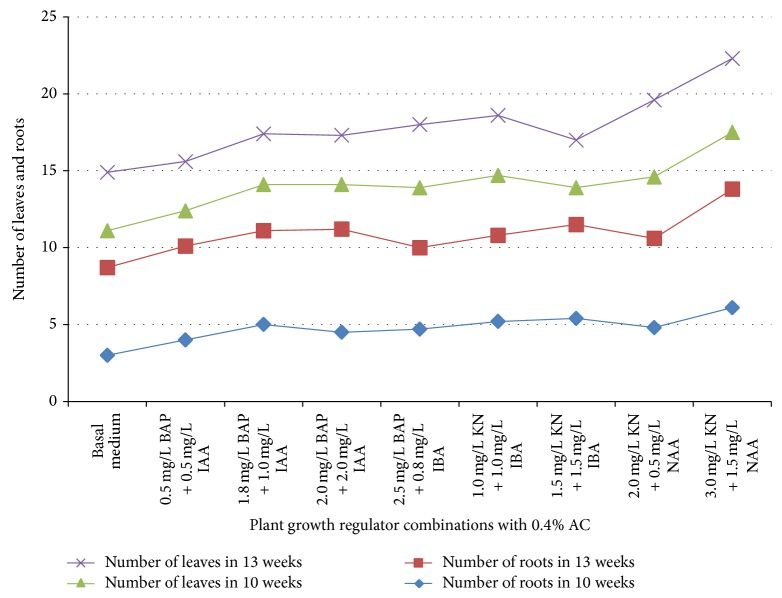
Comparison of* in vitro* leaf and root formation in seedlings of 10 and 13 weeks regenerated on medium supplemented with different combinations and concentrations of plant growth regulators.

Specific hormone combinations and concentrations are critical for inducing shoot multiplication and leaf proliferation in* D. chrysotoxum*. Arditti and Ernst [26] indicated the importance of exogenous supply of auxins and/or cytokinins for shooting initiation, leaf multiplication, and plantlet regeneration in orchids. Combination of BAP with IAA or IBA and KN with IAA or NAA resulted in high shooting induction and leaf multiplication (Figure 1(d)). The medium augmented with combination of 2.0 mg/L KN and 0.5 mg/L NAA induced maximum leaf formation (4.0 ± 1.4) and also leaf production did not differ significantly for other combinations M + 2.5 mg/L BAP + 0.8 mg/L IBA; M + 1.0 mg/L KN + 1.0 mg/L IBA; and M + 3.0 mg/L KN + 1.5 mg/L NAA (Table 2). Several authors have mentioned production of good shooting response and leaf formation under the influence of combinations of cytokinins and auxins in* Vanda spathulata* [27],* Cattleya* [28],* Cymbidium aloifolium* [29], and* Cymbidium bicolor* [30]. Rooting response in* D. chrysotoxum* in the present study was high for all the combinations investigated except for basal M medium with no growth regulators. The root number recorded was lower (3.0 ± 1.3) in basal medium with AC as compared to other combinations containing auxins and cytokinins at different concentrations. Low rooting response in basal medium and increasing influence of IAA, IBA, and NAA on higher root formation were similarly observed in* Dendrobium chrysotoxum* Lindl. [31]. In culture duration of 10 weeks, the highest root number (6.1 ± 1.4) was recorded in medium supplemented with 3.0 mg/L KN and 1.5 mg/L NAA (Table 2). The stimulatory effect of auxins in rooting initiation and multiplication had previously been reported in several orchid species [32–35]. The seedlings after 10 weeks were subcultured to similar culture conditions with same plant growth regulator combinations. There was significant rise in leaf and root number per plantlet and their corresponding length in basal M, M + 0.5 mg/L BAP + 0.5 mg/L IAA, M + 2.5 mg/L BAP + 0.8 mg/L NAA, M + 1.0 mg/L KN + 1.0 mg/L IBA, M + 2 mg/L KN + 0.5 mg/L NAA, and M + 3 mg/L KN + 1.5 mg/L NAA in just three weeks after the transfer (Table 3). However, the increase in leaf and root formation was marginal in M + 1.5 mg/L BAP + 1.0 mg/L IAA, M + 2.0 mg/L BAP + 2 mg/L IAA, and M + 1.5 mg/L KN + 1.5 mg/L IAA (Figure 2).

The influence of culture conditions in leaf and root formation was also investigated further by transferring the plantlets from initial culture conditions to newly prepared medium with different plant growth regulator combinations and concentrations. The leaf and root number and their corresponding length in new culture environment were depicted in Table 4. Shifting of seedlings from initial combination of M + 0.5 mg/L IAA + 0.5 mg/L BAP to fresh medium with 0.8 mg/L IBA + 2.5 mg/L BAP did not produce significant change in root and leaf number. When seedlings from M + 2.5 mg/L BAP + 0.8 mg/L IBA were transferred to 0.5 mg/L BAP + 3.0 mg/L IBA, there was reduction in shoot number (from 4.1 ± 1.1 to 3.4 ± 0.4), though root number increased sharply (from 5.3 ± 0.9 to 13.2 ± 1.3) in new combination (Figure 1(e)). However, when seedlings were planted on medium with lesser concentration of 0.5 mg/L IBA and higher 3.5 mg/L BAP content from the initial condition, shoot formation was pronounced, though there was slight increase in root number (Table 4). The above observations suggest the differential influence of IBA and BAP on root and shoot formation when present together in higher concentration. This finding is in agreement with the earlier reports of* in vitro* rooting and leaf formation in orchids under the influence of differential concentration of auxins and cytokinins [22, 36]. Transplanting of seedlings from initial combination of M + 1.0 mg/L KN + 1.0 mg/L IBA to medium with only 4.5 mg/L NAA greatly enhanced rooting development from the initial root number of 3.9 ± 1.6 to 11.1 ± 2.1 roots in new combination. When plantlets from M + 2.0 mg/L KN + 0.5 mg/L NAA were tested in new medium with increased concentration of 3.5 mg/L KN alone, there was no significant difference in leaf development as the number of leaves recorded remained the same even after three weeks of transfer to new combination. Similar response of slow leaf formation was observed when plantlets generated in M + 3.0 mg/L KN + 1.5 mg/L NAA were shifted to medium containing only 3.5 mg/L KN. This indicates the weak influence of KN on shooting and leaf formation in* D. chrysotoxum* when the growth hormone is not associated with auxins. The regenerated plantlets with healthy leaves and roots were hardened by growing them initially in basal medium with brick, charcoal pieces, and coconut fibres in similar ratios for about 3 weeks (Figure 1(f)). The plantlets were taken out from the culture vessels and transferred to small plastic pots with brick pieces, pine bark, charcoal pieces, and moss (1 : 1 : 1) as potting mixture. The transplanted plants were successfully acclimatized in glass house for 6–8 weeks before they were shifted to field conditions. There is no restriction in the number of plantlet production using plant tissue culture techniques when there are no limiting conditions and more than thousands of* Dendrobium chrysotoxum* plantlets can be possibly produced using the standardized mass multiplication protocol. Considering its high ornamental and medicinal values, the propagation of this orchid in larger scale will generate good income for local orchid farmers.

## 4. Conclusion

The present investigation brings forward the exciting revelations of varied* in vitro* cultural behaviour of seed explant in terms of seed germination, callus, shoot, and root development in response to different culture conditions. In ten different hormonal combinations tested, the best germination response was observed in Mitra medium supplemented with 2.0 mg/L BAP and 2 mg/L IAA. While highest leaf formation for 13 weeks old culture was recorded in medium enriched with 2.0 mg/L KN and 0.5 mg/L NAA, the maximum rooting response was induced in 3.0 mg/L KN and 1.5 mg/L NAA integrated medium. The enrichment of medium with higher concentration of IBA or NAA along with low cytokinins content induced excellent rooting response in the culture. BAP and KN on the other hand produced more pronounced shooting multiplication and leaf formation when associated with lower concentration of auxins rather than existing singly. The* in vitro* seed culture protocol established from the present study can be employed for large scale propagation of this medicinally important multiutility* Dendrobium *orchid for commercial and conservation purposes.

## Figures and Tables

**Table 1:** *In vitro *seed germination response of* Dendrobium chrysotoxum *on Mitra medium supplemented with different plant growth regulators.

BAP (mg/L)	KN (mg/L)	IAA (mg/L)	IBA (mg/L)	NAA (mg/L)	Activated charcoal (%)	Seed germination percentage	Germinated seeds developing into plantlets after 8 weeks (%)	Callus response∗∗∗
0	0	0	0	0	0.4	82.4 ± 3.4^e^	75.01 ± 5.1^d^	+++
0.5		0.5			0.4	90.2 ± 2.7^d^	82.35 ± 4.6^c^	+++
1.8		1.0			0.4	93.3 ± 4.8^c^	90.44 ± 6.2^b^	+++
2.0		2.0			0.4	98.1 ± 3.9^b^	91.63 ± 3.7^b^	++++
2.5			0.8		0.4	96.1 ± 4.3^a^	82.22 ± 4.3^b^	+++++
	1.0		1.0		0.4	93.5 ± 2.7^a^	87.05 ± 2.8^a^	++++
	1.5		1.5		0.4	95.2 ± 3.9^a^	92.66 ± 3.2^b^	++
	2.0			0.5	0.4	95.1 ± 2.5^a^	86.47 ± 5.3^a^	++++
	3.0			1.5	0.4	96.4 ± 3.6^a^	87.20 ± 4.8^a^	++++

The results are based on 8 replicates per treatment in three repeated experiments. ± indicates the standard deviation values. Means followed by same letter are not significantly different at *P* = 0.05.

∗∗∗++: poor; +++: average; ++++: good; +++++: high rate.

**Table 2:** Effect of different plant growth regulators on the *in vitro* leaf and root formation of *Dendrobium chrysotoxum* in 10 weeks.

BAP (mg/L)	KN (mg/L)	IAA (mg/L)	IBA (mg/L)	NAA (mg/L)	Activated charcoal (%)	Number of leaves/plantlets	Leaf length (cm)	Number of roots/plantlets	Root length (cm)
0	0	0	0	0	0.4	2.4 ± 0.7^c^	0.3 ± 0.08^c^	3.0 ± 1.3^g^	0.4 ± 0.1^c^
0.5		0.5			0.4	2.3 ± 0.5^c^	0.5 ± 0.04^a^	4.0 ± 1.8^f^	1.2 ± 0.4^b^
1.8		1.0			0.4	3.0 ± 0.9^d^	0.5 ± 0.05^a^	5.0 ± 1.0^e^	1.2 ± 0.3^b^
2.0		2.0			0.4	2.9 ± 1.0^d^	0.4 ± 0.06^b^	4.5 ± 1.4^d^	1.1 ± 0.9^b^
2.5			0.8		0.4	3.9 ± 1.1^a^	0.5 ± 0.07^a^	4.7 ± 1.3^b^	1.2 ± 0.3^b^
	1.0		1.0		0.4	3.9 ± 0.9^a^	0.4 ± 0.09^b^	5.2 ± 1.5^c^	1.3 ± 0.4^a^
	1.5		1.5		0.4	2.4 ± 0.8^c^	0.4 ± 0.04^b^	5.4 ± 1.2^c^	1.4 ± 0.1^c^
	2.0			0.5	0.4	4.0 ± 1.4^b^	0.5 ± 0.08^a^	4.8 ± 1.3^b^	1.2 ± 0.1^b^
	3.0			1.5	0.4	3.7 ± 1.2^a^	0.5 ± 0.06^a^	6.1 ± 1.4^a^	1.3 ± 0.7^a^

The results are based on 8 replicates per treatment in three repeated experiments. ± indicates the standard deviation values. Means followed by same letter are not significantly different at *P* = 0.05.

**Table 3:** Effect of different plant growth regulators on the *in vitro* leaf and root formation of *Dendrobium chrysotoxum* in 3 weeks after first subculture ^**^.

BAP (mg/L)	KN (mg/L)	IAA (mg/L)	IBA (mg/L)	NAA (mg/L)	Activated charcoal (%)	Number of leaves/plantlets	Leaf length (cm)	Number of roots/plantlets	Root length (cm)
0	0	0	0	0	0.4	3.8 ± 0.8^d^	0.4 ± 0.2^d^	5.7 ± 2.9^b^	1.2 ± 1.0^e^
0.5		0.5			0.4	3.2 ± 1.0^c^	0.8 ± 0.2^b^	6.1 ± 2.4^c^	3.2 ± 0.1^d^
1.8		1.0			0.4	3.3 ± 0.8^c^	0.7 ± 0.6^b^	6.1 ± 2.6^c^	3.1 ± 0.9^d^
2.0		2.0			0.4	3.2 ± 0.8^c^	0.7 ± 0.4^b^	6.7 ± 0.5^e^	3.0 ± 1.2^d^
2.5			0.8		0.4	4.1 ± 1.1^e^	1.0 ± 0.3^a^	5.3 ± 0.9^d^	2.0 ± 0.9^c^
	1.0		1.0		0.4	3.9 ± 1.6^d^	0.6 ± 0.2^c^	5.6 ± 2.1^b^	3.6 ± 0.8^a^
	1.5		1.5		0.4	3.1 ± 0.8^c^	0.7 ± 0.2^b^	6.1 ± 1.9^c^	3.8 ± 0.6^a^
	2.0			0.5	0.4	5.0 ± 0.9^b^	1.1 ± 0.2^a^	5.8 ± 1.1^b^	2.8 ± 1.2^b^
	3.0			1.5	0.4	4.8 ± 0.9^a^	0.9 ± 0.6^a^	7.7 ± 0.8^a^	3.7 ± 1.4^a^

The results are based on 8 replicates per treatment in three repeated experiments. ± indicates the standard deviation values. Means followed by same letter are not significantly different at *P* = 0.05.

^**^First subculture was performed at 10 weeks from date of inoculation.

**Table 4:** Effect of different plant growth regulators on leaf and root formation after seedlings transferred from initial to new plant growth regulator combinations in 2 weeks after 4th subculture ^**^.

Initial PGR combinations with 0.4% AC		New PGR combinations with 0.4% AC	Number of leaves/plantlets	Leaf length (cm)	Number of roots/plantlets	Root length (cm)
0.5 mg/L IAA + 0.5 mg/L BAP	→	0.8 mg/L IBA + 2.5 mg/L BAP	3.8 ± 1.2^e^	1.4 ± 0.9^c^	6.8 ± 1.2^e^	2.1 ± 1.1^e^
2.5 mg/L BAP + 0.8 mg/L IBA	→	0.5 mg/L BAP + 3.0 mg/L IBA	3.4 ± 0.4^b^	1.3 ± 0.4^d^	13.2 ± 1.3^d^	4.4 ± 1.7^d^
	→	3.5 mg/L BAP + 0.5 mg/L IBA	5.7 ± 1.0^d^	2.5 ± 0.7^a^	7.5 ± 1.8^c^	2.5 ± 0.8^b^
1.0 mg/L KN + 1.0 mg/L IBA	→	4.5 mg/L NAA	3.4 ± 0.2^b^	1.5 ± 0.6^c^	11.1 ± 2.1^b^	4.6 ± 0.4^c^
2.0 mg/L KN + 0.5 mg/LNAA	→	3.5 mg/L KN	4.1 ± 0.3^c^	2.1 ± 0.3^b^	7.3 ± 1.2^c^	1.6 ± 0.7^a^
3.0 mg/L KN + 1.5 mg/L NAA	→	2.5 mg/L KN + 2.5 mg/L NAA	3.4 ± 0.9^b^	2.4 ± 0.4^a^	12.0 ± 1.2^b^	2.6 ± 1.4^b^
	→	3.5 mg/L KN	4.2 ± 0.7^a^	2.3 ± 0.6^a^	9.0 ± 1.6^a^	1.6 ± 0.8^a^

The results are based on 8 replicates per treatment in three repeated experiments. ± indicates the standard deviation values. Means followed by same letter are not significantly different at *P* = 0.05.

^**^Fourth subculture was performed at 19 weeks from date of inoculation.
